# P-1442. Bivalent RSV Prefusion F-Based Subunit Vaccine Recipients with Chronic Kidney Disease Achieved High Neutralizing Titers One Month After Vaccination

**DOI:** 10.1093/ofid/ofaf695.1629

**Published:** 2026-01-11

**Authors:** Venkatesh Nadar, Jose F Cardona, Daniel P Eiras, Natalia Castillo-Almeida, Farah Rahman, Tarek Mikati, Vishva Bangad, John Woodside, Hayley Wyper, Maria Maddalena Lino, Elliot N DeHaan, Michael Patton, Kumar Ilangovan, Elena Kalinina, David Cooper, David Radley, Kena A Swanson, Annaliesa S Anderson, Alejandra C Gurtman, Iona Munjal

**Affiliations:** University of Pittsburgh Medical Center, Camp Hill, Pennsylvania; Indago Research & Health Center, Hialeah, Florida; Pfizer, Inc., Pearl River, NY; 4. Beth Israel Deaconess Medical Center, Boston, Massachusetts; Pfizer, New York, NY; 3. Pfizer, Inc., Vaccine Research & Development, Pearl River, NY; Pfizer, Inc., Pearl River, NY; Pfizer, New York, NY; Pfizer, Inc. Vaccine Research & Development, Marlow, England, United Kingdom; Pfizer, New York, NY; Pfizer, New York, NY; Pfizer, Vaccine Research and Development, Hurley, England, United Kingdom; Vaccine Research and Development, Pfizer, USA, Raleigh, North Carolina; Pfizer, Inc., Pearl River, NY; Pfizer, New York, NY; Pfizer, New York, NY; Pfizer, New York, NY; Pfizer, New York, NY; Pfizer, New York, NY; Pfizer Inc

## Abstract

**Background:**

Chronic kidney disease (CKD) is associated with a higher incidence of severe respiratory syncytial virus (RSV) disease leading to hospitalizations, making individuals with CKD particularly vulnerable. Pfizer’s bivalent RSV prefusion F-based subunit vaccine (RSVpreF) has been approved in the United States for the prevention of lower respiratory tract disease (LRTD) caused by RSV in individuals aged 60 years and older, as well as in adults 18 to 59 who are at increased risk for RSV associated-LRTD. The immunogenicity of RSVpreF has been evaluated in healthy adults or those with chronic stable disease aged 60 years and older in the pivotal RENOIR C3671013 efficacy study, as well as in high-risk or renally-impaired adults aged 18 years and older in the MONeT C3671023 study. This analysis aims to evaluate the safety and immunologic response of RSVpreF vaccination in a subset of participants enrolled in both studies with a prior diagnosis of CKD (stages 1 to 5).

**Figure 1:**
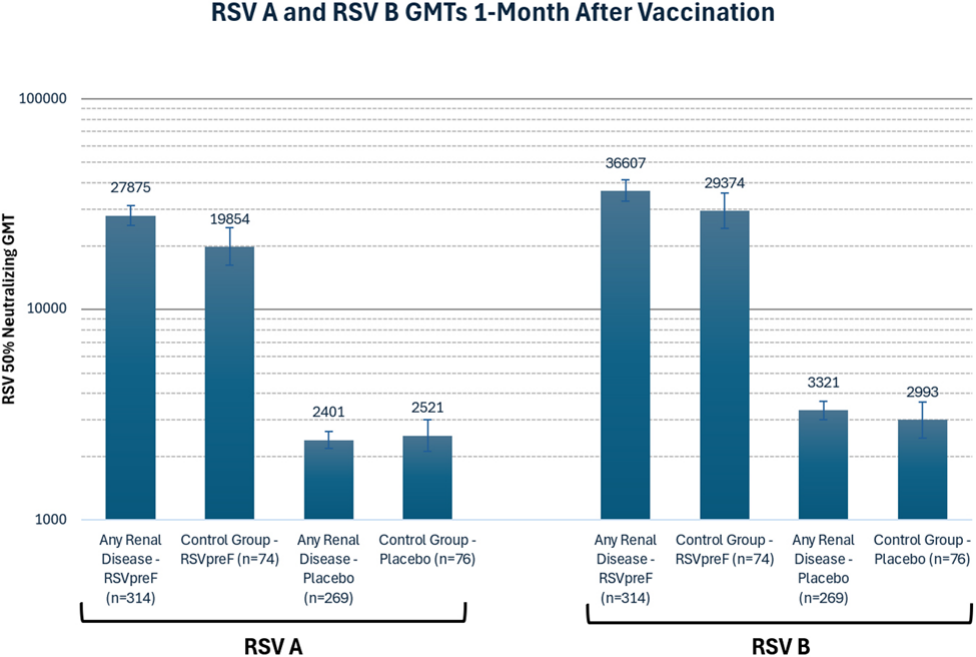
RSV A and RSV B Neutralizing GMTs 1-Month After Vaccination

**Table 1:**
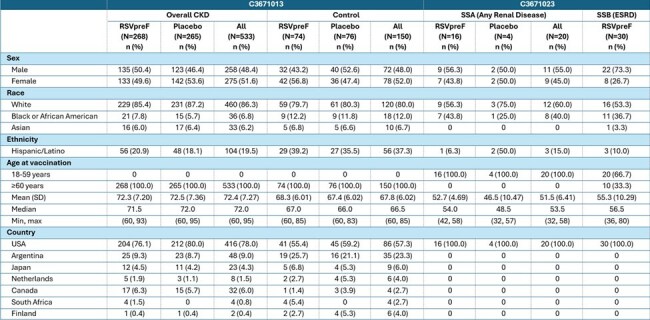
Demographic and Baseline Characteristics

**Methods:**

Participants with CKD from the RENOIR study and those with renal disease from the MONeT substudy A (SSA), as well as individuals with end-stage renal disease (ESRD) on hemodialysis (HD) from substudy B (SSB) were identified. Blood samples were analyzed for RSV A and B neutralizing geometric mean titers (GMTs). A randomly selected control group without CKD or HIV was selected from the RENOIR study. Safety was assessed in all subjects who met the selection criteria.

**Table 2:**
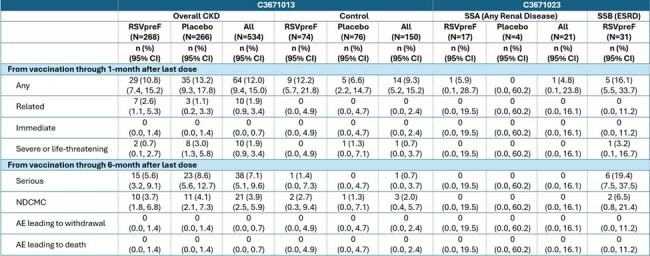
Adverse Events, by Analysis Interval and Category – Safety Population

**Results:**

RSV neutralizing titers (NT) in the CKD group were robust among those who received RSVpreF with GMTs of 27,875 for RSV A and 36,607 for RSV B at one-month postvaccination. In comparison, placebo recipients had GMTs of 2,401 for RSV A and 3,321 for RSV B. The NTs observed in the CKD group were comparable to those in the control group (RSV A and B GMTs 19,854 and 29,374, respectively) and those described previously from the RENOIR and MONeT earlier studies (Figure 1). Additionally, the CKD group experienced a higher frequency of related and serious adverse events (Table 2).

**Conclusion:**

Vaccination with bivalent RSVpreF elicited a strong immune response to both RSV A and B in adults with CKD stages 1 to 5 including those with ESRD on HD. No safety signals were identified in the CKD group.

**Disclosures:**

Daniel P. Eiras, MD, MPH, Pfizer, Inc.: Salary|Pfizer, Inc.: Stocks/Bonds (Public Company) Farah Rahman, DO, Pfizer, Inc.: Salary|Pfizer, Inc.: Stocks/Bonds (Public Company) Tarek Mikati, MD,MPH, Pfizer, Inc.: Salary|Pfizer, Inc.: Stocks/Bonds (Public Company) Vishva Bangad, MS, Pfizer, Inc.: Salary|Pfizer, Inc.: Stocks/Bonds (Public Company) John Woodside, PhD, Pfizer, Inc.: Salary|Pfizer, Inc.: Stocks/Bonds (Public Company) Hayley Wyper, BBiomedSc, Pfizer, Inc.: Salary|Pfizer, Inc.: Stocks/Bonds (Public Company) Maria Maddalena Lino, PhD, Pfizer, Inc.: Salary|Pfizer, Inc.: Stocks/Bonds (Public Company) Elliot N. DeHaan, MD, Pfizer, Inc.: Salary|Pfizer, Inc.: Stocks/Bonds (Public Company) Michael Patton, B.Sc., Pfizer, Inc.: Salary|Pfizer, Inc.: Stocks/Bonds (Public Company) Kumar Ilangovan, MD, MSPH, MMCi, Pfizer, Inc.: Salary|Pfizer, Inc.: Stocks/Bonds (Public Company) Elena Kalinina, PhD, Pfizer, Inc.: Salary|Pfizer, Inc.: Stocks/Bonds (Public Company) David Cooper, PhD, Pfizer, Inc.: Salary|Pfizer, Inc.: Stocks/Bonds (Public Company) David Radley, MS, Pfizer, Inc.: Salary|Pfizer, Inc.: Stocks/Bonds (Public Company) Kena A. Swanson, Ph.D., Pfizer, Inc.: Salary|Pfizer, Inc.: Stocks/Bonds (Public Company) Annaliesa S. Anderson, PhD, Pfizer Inc: Employee Alejandra C. Gurtman, M.D., Pfizer, Inc.: Salary|Pfizer, Inc.: Stocks/Bonds (Public Company) Iona Munjal, MD, Pfizer, Inc.: Salary|Pfizer, Inc.: Stocks/Bonds (Public Company)

